# Intussusception of the appendix in a young adult: an important differential diagnosis of abdominal pain in cystic fibrosis patients?

**DOI:** 10.1093/jscr/rjad117

**Published:** 2023-03-17

**Authors:** Meena Venkatasami, Ellen Cobby

**Affiliations:** Department of Pathology, Royal Shrewsbury Hospital, Shrewsbury SY3 8XQ, UK; Department of Pathology, Royal Shrewsbury Hospital, Shrewsbury SY3 8XQ, UK

**Keywords:** Intussusception, appendix, distal intestinal obstructive syndrome, cystic fibrosis

## Abstract

Cystic fibrosis (CF) is commonly associated with gastrointestinal manifestations from infancy to adulthood. Distal intestinal obstruction syndrome (DIOS) affects 20% of CF patients, where intussusception can be a rare complication. A 20-year-old CF male was diagnosed with a 3-day history of right iliac fossa pain and diarrhoea. Clinical examination revealed a tender palpable mass in the right iliac fossa and raised serum inflammatory markers. Contrast computerized-tomography of the abdomen-pelvis suggested intussusception of the appendix and further confirmed on histological analyses. The patient underwent an open appendicectomy where the intussusception had self-resolved. The literature review indicated a scarcity of data with 10 cases reported of intussusception in adult CF patients. Our case was in line with previous research of transient intussusception. This rare case highlights an importance to carry a higher index of suspicion for gastrointestinal manifestations in CF patients where differential diagnoses of DIOS and intussusception should be considered in the acute presentation.

## INTRODUCTION

Cystic fibrosis (CF) is commonly associated with gastrointestinal manifestations from birth to adulthood. Patients with CF have an increased risk of intestinal disorders due to pancreatic insufficiency, resulting in malabsorption, mucous thickening and slowing gastrointestinal transit [1]. Such disorders include intussusception, a phenomenon whereby a segment of bowel invaginates intraluminally into an adjacent segment of bowel. Clinical presentation classically includes abdominal pain, nausea and vomiting, constipation, melaena and fever [2].

In children, intussusception is uncommon affecting 1% of patients with CF, usually within the first couple of years of life [1–3]. This differs from the adult population, where intussusception may develop as a rare complication from the more common distal intestinal obstruction syndrome (DIOS), which affects up to 20% of patients with CF (of which half will have pancreatic insufficiency) [2]. Intussusception in adults with CF is very rare (1%), and appendiceal intussusception in adults with CF is scarcely reported at all in the literature [1].

The pathophysiology of DIOS is poorly understood but thought to occur most frequently in the distal ileum, which becomes either completely or partially blocked with mucinoid secretions, thereby providing a lead point for intussusception [1]. DIOS is common in CF patients due to their malabsorptive predisposition, resulting feculent thickening and slow gastrointestinal transit [1]*.* Some cases may present as the acute abdomen, with non-specific symptoms including abdominal pain, cramping and constipation, which may mask under common differential diagnoses like appendicitis and inflammatory bowel disease, the management of which is very different from intussusception.

## CASE DESCRIPTION

We report a case of a 20-year-old male with CF, who was admitted to the emergency department with a 3-day history of intermittent right iliac fossa pain and diarrhoea. He presented with no nausea or vomiting and had no history of previous abdominal surgery. The patient was taking regular CF-related medications including Creon for CF-related pancreatic insufficiency. On admission, the patient was afebrile and hypotensive, requiring intravenous fluid resuscitation. Clinical examination revealed a soft abdomen, with a tender, palpable mass in the right iliac fossa and present bowel sounds. Serum laboratory investigations revealed a mild leucocytosis of 11.8 × 10^9^/L, and raised CRP of 53 mg/L, with amylase (60 IU/L), liver function tests and lactate within normal ranges. At this stage, the provisional diagnosis was acute appendicitis and further imaging was requested.

**Figure 1:**
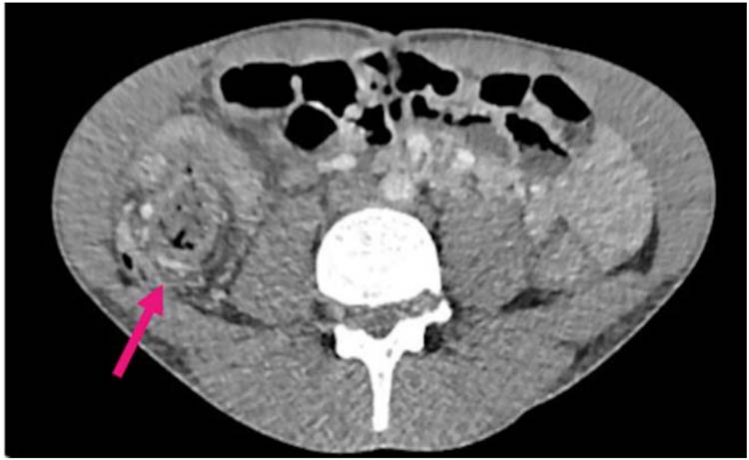
Contrast CT axial sections showing the enhanced thickened appendix (white) intussuscepts with fat stranding, into the ascending colon. Distal faecal loading also observed.

**Figure 2:**
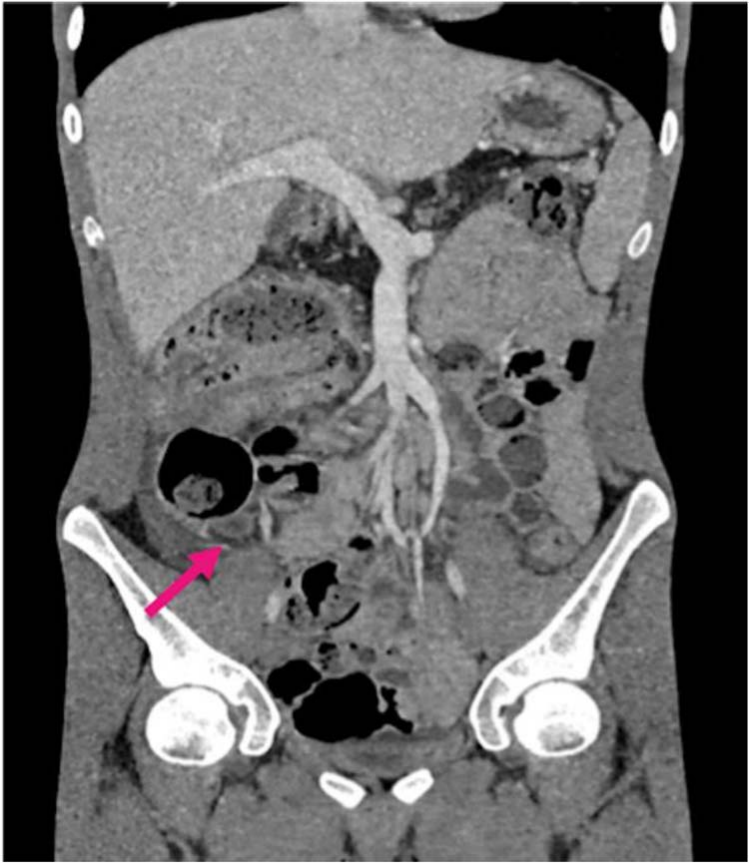
Contrast CT coronal sections depicting the classical ‘bullseye sign’ of intussusception of the caecum into the ascending colon.

Radiological investigations included a contrast computerized-tomography (CT) of the abdomen/pelvis and suggestive of intussusception of the caecum into the ascending colon, with a thickened appendix. Due to the presence of faecal loading, it was not clear radiologically whether the combined clinical picture was suggestive of distal intestinal syndrome (Figs 1 and 2) due to faecal loading only, or true intussusception of the appendix.

The patient’s symptoms did not improve upon admission and thus underwent an emergency laparotomy-open appendicectomy the same day. Interestingly, by the time of the procedure, intraoperative findings showed the intussusception spontaneously resolved, no longer requiring surgical reduction. The appendix appeared dilated and distended and thus surgically removed.

Histological analysis of the appendix demonstrated a non-neoplastic specimen, with hard, impacted intraluminal mucinous material confirming a diagnosis of appendiceal intussusception. Focal neutrophilic infiltration of the surface epithelium was present, but interestingly, there was no transmural acute inflammation typically seen in acute appendicitis. There was also dilatation of crypts containing mucin reported, characteristic in CF.

During the immediate postoperative period, the patient’s abdominal symptoms improved on the ward. The patient’s recovery was complicated by a decline in respiratory function on day 3 of recovery, with a new oxygen requirement. Chest radiography confirmed a pneumonia, for which he was transferred to a specialist CF centre for further respiratory management. The patient made a full recovery and discharged a few days later.

## DISCUSSION

Literature review shows a scarcity of research with only 10 cases of intussusception in adult CF patients reported [4]. To our knowledge, only two cases of appendiceal intussusception in CF adults have been reported, in patients aged 18 and 19 years old, respectively, suggesting a predilection for young adults [5, 6].

Intussusception may present in a similar fashion to DIOS; with crampy abdominal pain, a palpable mass and vomiting as a late sign due to obstruction. These overlapping signs and symptoms and make the differential diagnosis of intussusception particularly challenging to discern in the acute presentation. Therefore, it is crucial to undertake appropriate radiological investigation to optimize diagnosis and management for the patient.

As demonstrated in our case, CT offers high diagnostic yield for complications of CF in the acute setting (78%) but is not always sensitive for intussusception [7]. Other imaging modalities include ultrasound, used commonly in paediatrics, which demonstrate the Bull’s eye sign of intussusception [8]. This, however, may have limited diagnostic yield in adults and has the additional caveat of poor availability compared to CT scanning out-of-hours.

More recent studies report the use of colonoscopy for diagnosis of intussusception in patients with CF, where a diagnosis has been difficult to deduce [5].

Interestingly, the intussusception in our case had reduced by the time of operation, and surgical reduction was no longer warranted. This is in line with previous case reports in the adult CF population, where the intussusception has been transient and resolved spontaneously [9, 10]. In this case presentation, surgery was indicated following radiological imaging, to prevent bowel ischaemia by restoring blood flow to the affected bowel segment [11].

In the literature, one case report included the safe use of contrast enema for the treatment of intussusception of an adult male with CF, a therapy that is commonly used to treat intussusception in children [12, 13]. Overall, there are insufficient data in the literature to draw any standardized therapeutic protocol, but this could be further investigated empirically, ideally with a multicentre collaboration due to the rarity of the presentation.

## CONCLUSION

From this rare case presentation of appendiceal intussusception in a CF adult, we have learnt it is imperative to carry a higher index of suspicion of gastrointestinal complications in CF patients. Acute appendicitis is seen less often in this patient cohort and differentials such as DIOS and intussusception should be considered, where rapid surgical exploration and reduction is considered the mainstay of treatment.
